# Host genetic variation explains reduced protection of commercial vaccines against *Piscirickettsia salmonis* in Atlantic salmon

**DOI:** 10.1038/s41598-020-70847-9

**Published:** 2020-10-26

**Authors:** Carolina Figueroa, Pamela Veloso, Lenin Espin, Brian Dixon, Débora Torrealba, Islam Said Elalfy, Juan Manuel Afonso, Carlos Soto, Pablo Conejeros, José A. Gallardo

**Affiliations:** 1 Escuela de Ciencias del Mar, Pontificia Universidad Católica de Valparaíso, Avenida Altamirano 1480, 2360007 Valparaíso, Región de Valparaíso Chile; 2 Programa de Doctorado en Ciencias, mención Recursos Naturales Acuáticos, Universidad de Valparaíso, Blanco 951, 2391415 Valparaíso, Región de Valparaíso Chile; 3 Instituto Universitario de Acuicultura Sostenible y Ecosistemas Marinos (IU-ECOAQUA), Grupo de Investigación en Acuicultura (GIA), Universidad de Las Palmas de Gran Canaria, Carretera de Taliarte, s/n, 35214, Telde, Spain; 4 Department of Biology, Faculty of Science, University of Waterloo, 200 University Ave W, Waterloo, ON N2L 3G1 Canada; 5 Department of Agricultural, Food & Nutritional Science, University of Alberta, 116 St & 85 Ave, Edmonton, AB T6G 2R3 Canada; 6 Salmones Camanchaca Diego Portales 2000, 5503642 Puerto Montt, Chile; 7 Centro de Investigación y Gestión de Recursos Naturales (CIGREN), Facultad de Ciencias, Universidad de Valparaíso, Blanco 951, 2391415 Valparaíso, Región de Valparaíso Chile

**Keywords:** Ichthyology, Ecological epidemiology, Heritable quantitative trait

## Abstract

Vaccination is a widely used control strategy to prevent *Piscirickettsia salmonis* causing disease in salmon farming. However, it is not known why all the currently available commercial vaccines generally fail to protect against this pathogenic bacteria. Here, we report, from two different populations, that between-family variation is a strong intrinsic factor that determines vaccine protection for this disease. While in some full-sib families, the protection added by vaccination increased the survival time in 13 days in comparison with their unvaccinated siblings; in other families, there was no added protection by vaccination or even it was slightly negative. Resistance to *P. salmonis,* measured as days to death, was higher in vaccinated than unvaccinated fish, but only a moderate positive genetic correlation was obtained between these traits. This disputes a previous hypothesis, that stated that both traits were fully controlled by the same genes, and challenges the use of unvaccinated fish as gold standard for evaluating and selecting fish resistant to *P. salmonis*, particularly if the offspring will be vaccinated. More studies are necessary to evaluate if variation in the host immune response to vaccination could explain the between-family differences in resistance observed in vaccinated fish.

## Introduction

Over the past few decades, aquaculture production has been growing steadily, to the point that it produces around 50% of the fish consumed globally today^1,2^. By 2020, it is predicted that aquaculture will be the prime source of fish protein in the world^3^. As in other livestock production systems, farmed fish are continually exposed to pathogens, including bacteria, viruses, and parasites, which may produce outbreaks and mortality^1,4^. Recently, it has been estimated that as much as 10% of all cultured aquatic animals are lost to infectious diseases, amounting to > 10 billion USD annual losses on a global scale^5^. When bacterial outbreaks occur, antibiotics are used to maintain fish health and productivity. However, excessive use of antibiotics is generating concern about public health^1,6^, due to its possible contribution to the spread of drug-resistant pathogens in both farmed animals and wild fish.

Prevention of diseases in aquaculture species includes the use of a diverse but traditional set of strategies and management solutions, including vaccines, immunostimulants, probiotics, and dietary supplements^7,8^. Vaccination is considered crucial as it is one of the most important approaches to preventing and controlling diseases in world aquaculture^8^. As a result, vaccines are available for 24 major infectious bacterial diseases, 12 viral infections, and a handful of ectoparasites of fish^9^. Nevertheless, the control of some bacterial and parasitic infections remains a significant problem affecting all major salmon producing countries, namely Norway, Canada, Scotland, and Chile^10^.

In the Chilean salmon industry, *Piscirickettsia salmonis* is the aetiological agent of piscirickettsiosis, a severe salmon disease that vaccines have failed to control^11,12^. *P. salmonis* causes necrosis of all lymphoid tissues and damages the liver, kidney, and spleen resulting in high mortality rates in different salmonid species including Rainbow trout, Pacific salmon, and Atlantic salmon^13,14^. Piscirickettsiosis has been infecting Chilean salmon for almost 40 years and is considered the main bacterial threat to this industry^1,15,16^. Currently, there are 32 vaccines commercially available against piscirickettsiosis from monovalent live attenuated vaccines to pentavalent inactivated vaccines, including *P. salmonis* bacterin ^17^. Failure of the vast majority of vaccines to protect against this bacterium has been proposed as the cause of the increased use of antibiotics in the Chilean farmed salmon industry^16^. The reason for the failure of the vaccines to provide protection against *P. salmonis* is currently unknown, but it has been proven that some extrinsic factors, such as coinfection with sea lice *Caligus rogercresseyi,* which is a highly prevalent parasite in Chile, override the protective effect of the vaccines^18^. Furthermore, it has been suggested that circulating levels of anti-*P. salmonis* antibodies decline only a few months after vaccination, thus fish could be more susceptible in their adult state^19^.

In this study, we investigated whether between-family variation of fish could intrinsically explain the reduced protection of commercial vaccines against *P. salmonis*. Through a set of analyses at the molecular, individual, family, and population level, we demonstrated that while some vaccinated families seem not to benefit from current vaccination strategies, others do. Due to the enormous impact of this pathogen on the health of the fish, the productivity of the Chilean salmon industry, and the high use of antibiotics, it is necessary to implement new and better fish health improvement strategies that do consider genetic variability of hosts.

## Results

### Between-population phenotypic variation of the resistance against *P. salmonis* and protection added by vaccination

**Figure 1:**
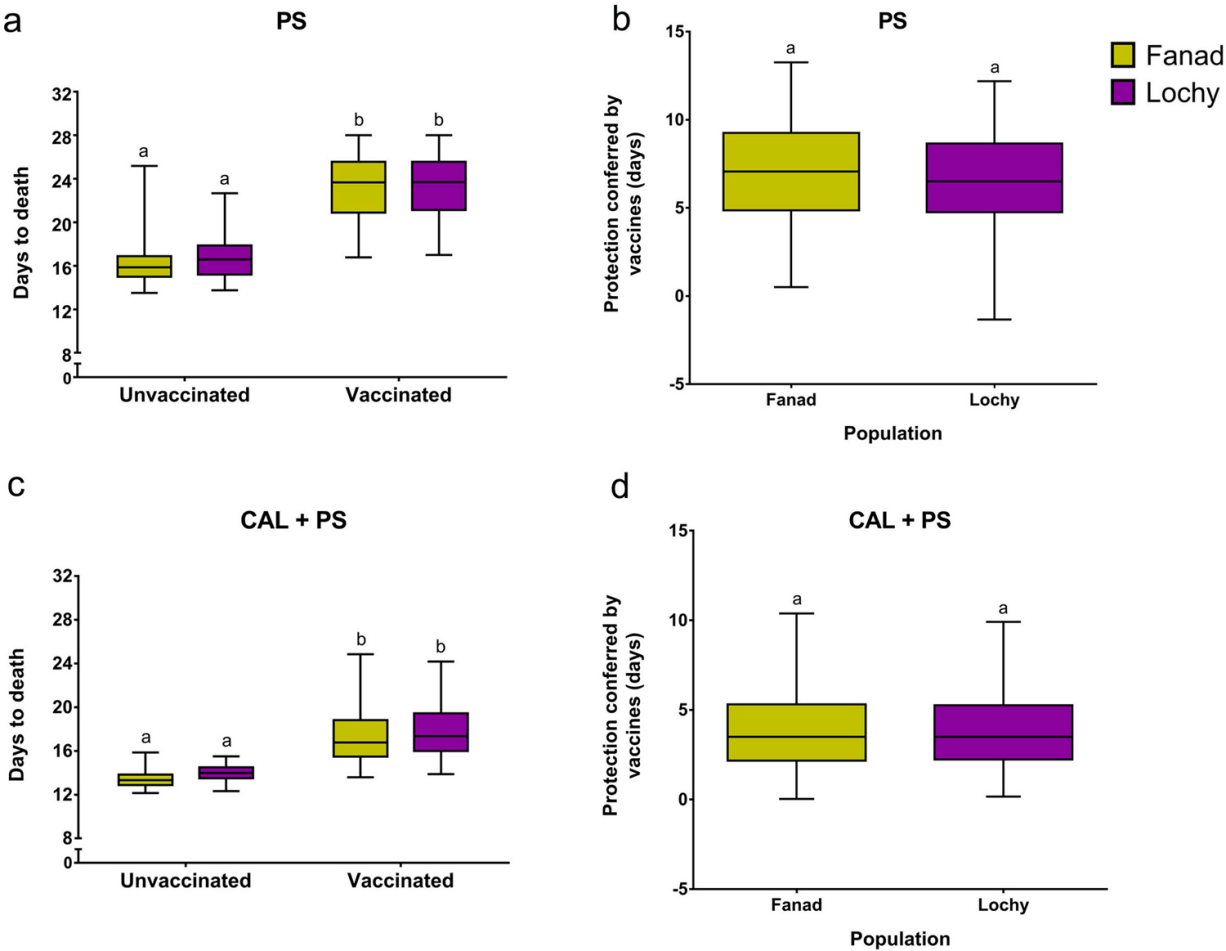
Differences in the resistance to *P. salmonis* and protection conferred by vaccines at the population level. Boxplots show days to death for vaccinated and unvaccinated families of the populations Fanad (yellow) and Lochy (purple) that were exposed to (**a**) a single infection with *P. salmonis* (PS) or (**b**) coinfection with *C. rogercresseyi* and *P. salmonis* (CAL + PS). Protection conferred by vaccines for fish families of both populations that were exposed to (**c**) a single infection with *P. salmonis* (PS) or (**d**) coinfection with *C. rogercresseyi* and *P. salmonis* (CAL + PS) are also shown as boxplots. Data represent mean ± SD. Significance levels were obtained using a two-way ANOVA followed by a Tukey post-hoc test and unpaired t-test.

To evaluate the resistance of Atlantic salmon to *P. salmonis* a disease challenge was performed in two domesticated populations of different origin, Fanad and Lochy. A total of 2,905 fish were challenged in Fanad and 2,876 fish in Lochy, belonging to 100 full-sib and 50 half-sib families for each population (Table 1). Fish were exposed to four different environments: with and without vaccination, with a single infection with *P. salmonis* (PS) and coinfection with *C. rogercresseyi* and *P. salmonis* (CAL + PS). Resistance was evaluated as days to death after the challenge with *P. salmonis*, which had a high family phenotypic correlation with the mortality measured as a binary trait (r_p_ ranging from − 0.72 to − 0.93). It suggests that both traits could be used interchangeably (Fig. S1 online). Resistance against *P. salmonis* in vaccinated fish and protection added by vaccination was not significantly different between populations (Fig. 1a,b). Differences between populations in the unvaccinated fish were not observed either.

**Table 1:** Total fish, average body weight, number of full and half-sib families, mean, minimum, and maximum of fish per family from each population.

Population	Treatment	Number of total fish	Body weight	Number of full-sib	Number of half-sib	Mean of fish per full-sib families	Min of fish per full-sib families	Max of fish per full-sib families
**Fanad**	Vaccinated	1,411	112.9 ± 28.0	100	50	14	10	17
	Unvaccinated	1,494	110.3 ± 26.8	100	50	15	10	17
	Total	2,905	111.6 ± 27.4	100	50	29	21	33
**Lochy**	Vaccinated	1,425	97.6 ± 22.9	100	50	14	6	17
	Unvaccinated	1,451	101.1 ± 22.4	100	50	15	9	17
	Total	2,876	99.4 ± 22.7	100	50	29	21	32

Increased protection against *P. salmonis* due to vaccination during coinfection with sea lice was also evaluated. The prevalence of sea lice was very high for both vaccinated (99.8%) and unvaccinated (99.7%) fish, and the average abundance of lice was not significantly different between the vaccinated and unvaccinated groups (29 ± 24 and 28 ± 24, respectively). The abundance of sea lice was higher in population Fanad than in population Lochy (lice per fish: mean _Fanad_ = 41.8 ± 1.3, mean _Lochy_ = 15.6 ± 0.6). Although, despite this particular difference, resistance against *P. salmonis* in vaccinated fish, coinfected with sea lice, and protection added by vaccination was not significantly different between populations (Fig. 1c,d). Similar to the single infection, differences were not observed between populations in the unvaccinated fish.

### Between-family phenotypic variation in resistance to *P. salmonis* and protection added by vaccination

**Figure 2:**
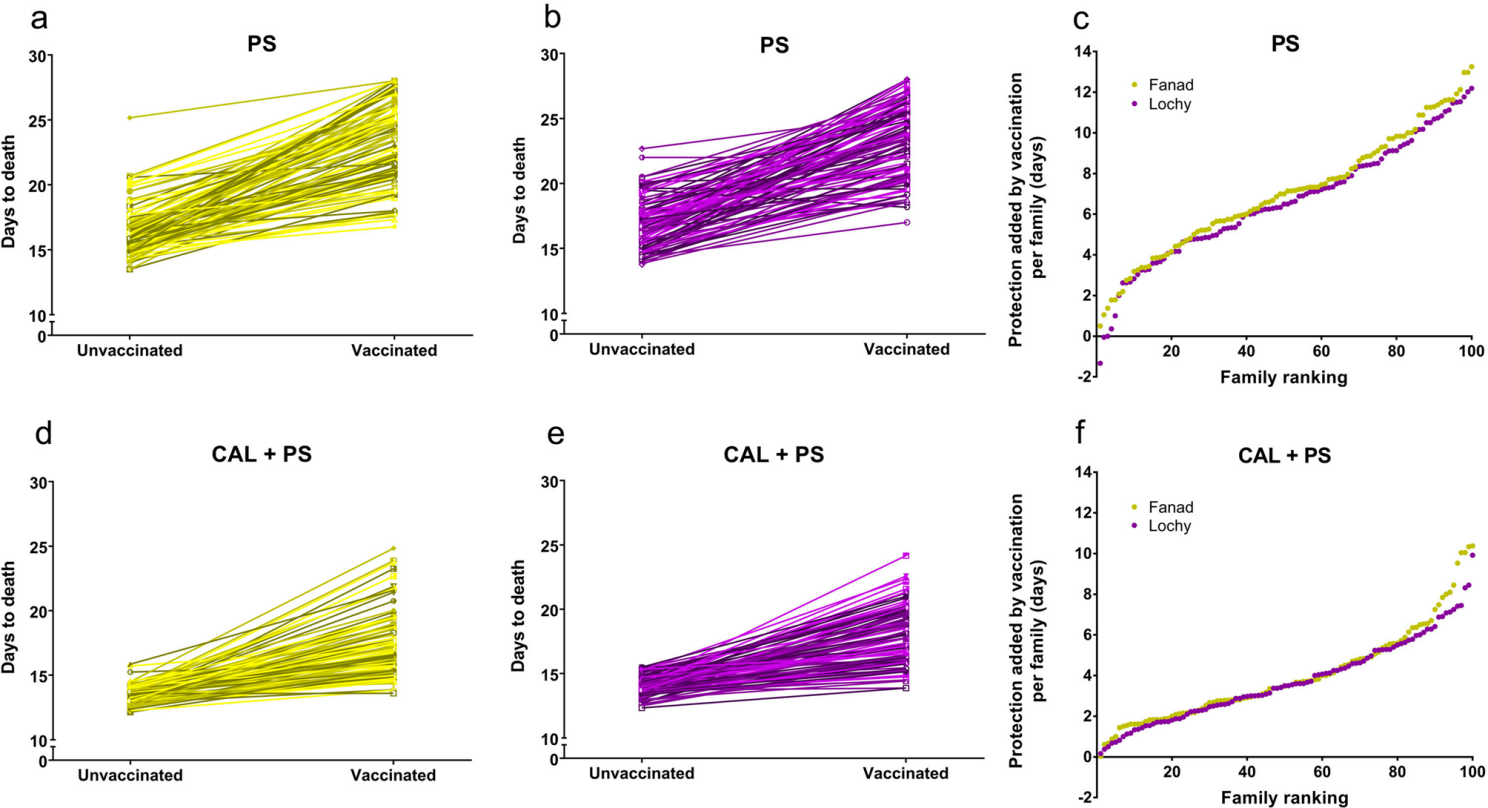
Family level changes in resistance to *P. salmonis* and protection conferred by vaccines. Changes in days to death between unvaccinated and vaccinated fish families of the population Fanad and Lochy that were exposed to (**a**,**b**) a single infection with *P. salmonis* (PS) or (**d**,**e**) coinfection with *C. rogercresseyi* and *P. salmonis* (CAL + PS) are shown. Also shown are the days of protection added by vaccination per family for both populations that were exposed to (**c**) a single infection with *P. salmonis* (PS) or (**f**) coinfection with *C. rogercresseyi* and *P. salmonis* (CAL + PS).

In both populations, the resistance against *P. salmonis* and the added protection by vaccination was very variable between families (Fig. 2a–c). Many vaccinated families showed similar or worse performance than that seen in the best-unvaccinated families. Notably, in some families, the protection added by vaccination was zero or even slightly negative when compared with their unvaccinated siblings. In contrast, in other families, vaccination added up to 13 days more survival time in a single infection. During coinfection, the resistance against *P. salmonis* and the protection added by vaccination was also highly variable between families (Fig. 2d–f); however, responses to coinfection also differed from a single infection. First, the phenotypic variation of days to death was lower in unvaccinated fish than in vaccinated fish, and second maximum protection added by vaccination decreased from 13 to 10 days in coinfection with sea lice.

### Heritability and genetic correlation of resistance in vaccinated and unvaccinated fish

**Figure 3:**
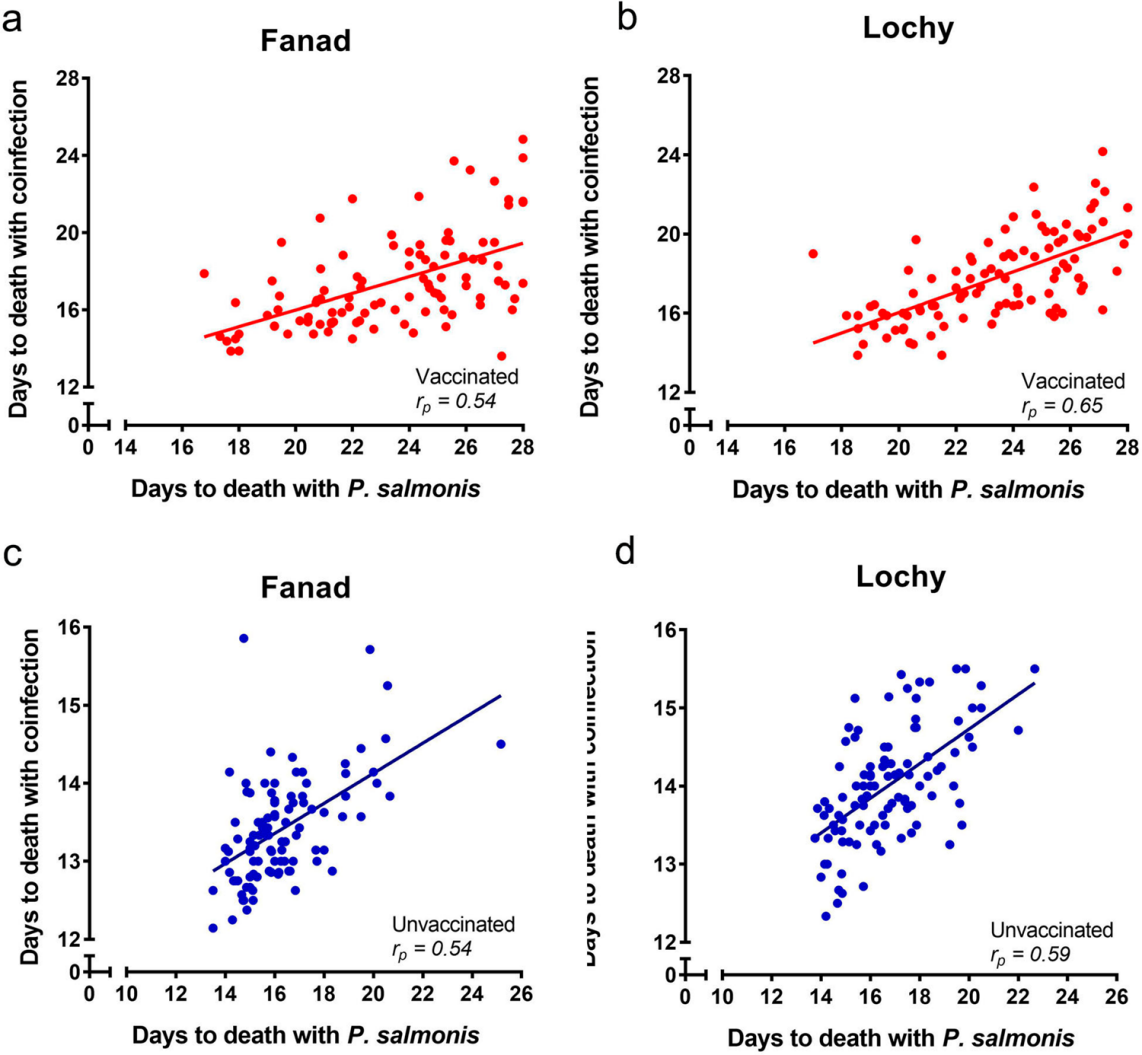
Familiar phenotypic correlations (r_p_) of the number of days to death in a single infection with *P. salmonis* and coinfection with *C. rogercresseyi *and *P. salmonis* in population Fanad (**a**: Vaccinated, **c**: Unvaccinated) and Lochy (**b**: Vaccinated, **d**: Unvaccinated).

Resistance to *P. salmonis* in both populations showed a moderate to high heritabilities (*h*^2^ ranging from 0.23 ± 0.06 to 0.65 ± 0.07, Table 2) in both infection scenarios for vaccinated and unvaccinated fish (Figs. S2, S3 online). The largest heritability was found in coinfected vaccinated fish (Table 2). On the other hand, genetic correlation between single infection and coinfection were very high and positive in every scenario ranging from 0.33 ± 0.12 to 0.99 ± 0.02 (Table 2, Fig. 3). However, the genetic correlation between the resistance of vaccinated and unvaccinated fish was of medium magnitude ranging from 0.76 ± 0.09 to 0.33 ± 0.12, suggesting that both traits are not fully controlled by the same genes (Table 2, Fig. 3). It was noteworthy that population Fanad displayed a higher estimation of heritability (three out of four) and genetic correlation than population Lochy (Table 2), showing that although there were no phenotypic differences at the population-level, genetic differences could be observed between the two populations.

**Table 2:** Heritability, genetic and phenotypic correlations of the trait days to death after a challenge with *P. salmonis* (PS) and coinfection with *C. rogercresseyi* and *P. salmonis* (CAL + PS) in Atlantic salmon estimated in vaccinated (V) and unvaccinated (U) fish from population Fanad and Lochy.

Population	U-PS	U-CAL + PS	V-PS	V-CAL + PS
**Fanad**				
U-PS	0.48 ± 0.06 **	*0.99* ± *0.02* **	*0.65* ± *0.10* **	*0.69* ± *0.09* **
U-CAL + PS	0.54 ± 0.07 **	0.23 ± 0.06 **	*0.65* ± *0.11* **	*0.76* ± *0.09* **
V-PS	0.33 ± 0.09 **	0.27 ± 0.09 **	0.38 ± 0.07 **	*0.88* ± *0.07* **
V-CAL + PS	0.43 ± 0.08 **	0.32 ± 0.09 **	0.54 ± 0.07 **	0.65 ± 0.07 **
**Lochy**				
U-PS	0.34 ± 0.06 **	*0.92* ± *0.08* **	*0.47* ± *0.13* **	*0.33* ± *0.12* *
U-CAL + PS	0.59 ± 0.07 **	0.26 ± 0.04 **	*0.51* ± *0.15* **	*0.60* ± *0.11* **
V-PS	0.23 ± 0.09 *	0.28 ± 0.09 **	0.36 ± 0.07 **	*0.93* ± *0.06* **
V-CAL + PS	0.15 ± 0.10 ns	0.32 ± 0.09 **	0.65 ± 0.06 **	0.50 ± 0.06 **

Heritabilities (in bold at the diagonal ± standard error) for the number of days to death; genetic correlations (in italics above the diagonal ± standard error) and phenotypic correlations (below the diagonal ± standard error).

*U* unvaccinated; *V* vaccinated; *PS* single infected with *P. salmonis*; *CAL + PS* coinfected with *C. rogercresseyi* and *P. salmonis*; .

ns: Non significant.

* P < 0.05; **P < 0.01.

### Comparison of bacterial load

**Figure 4:**
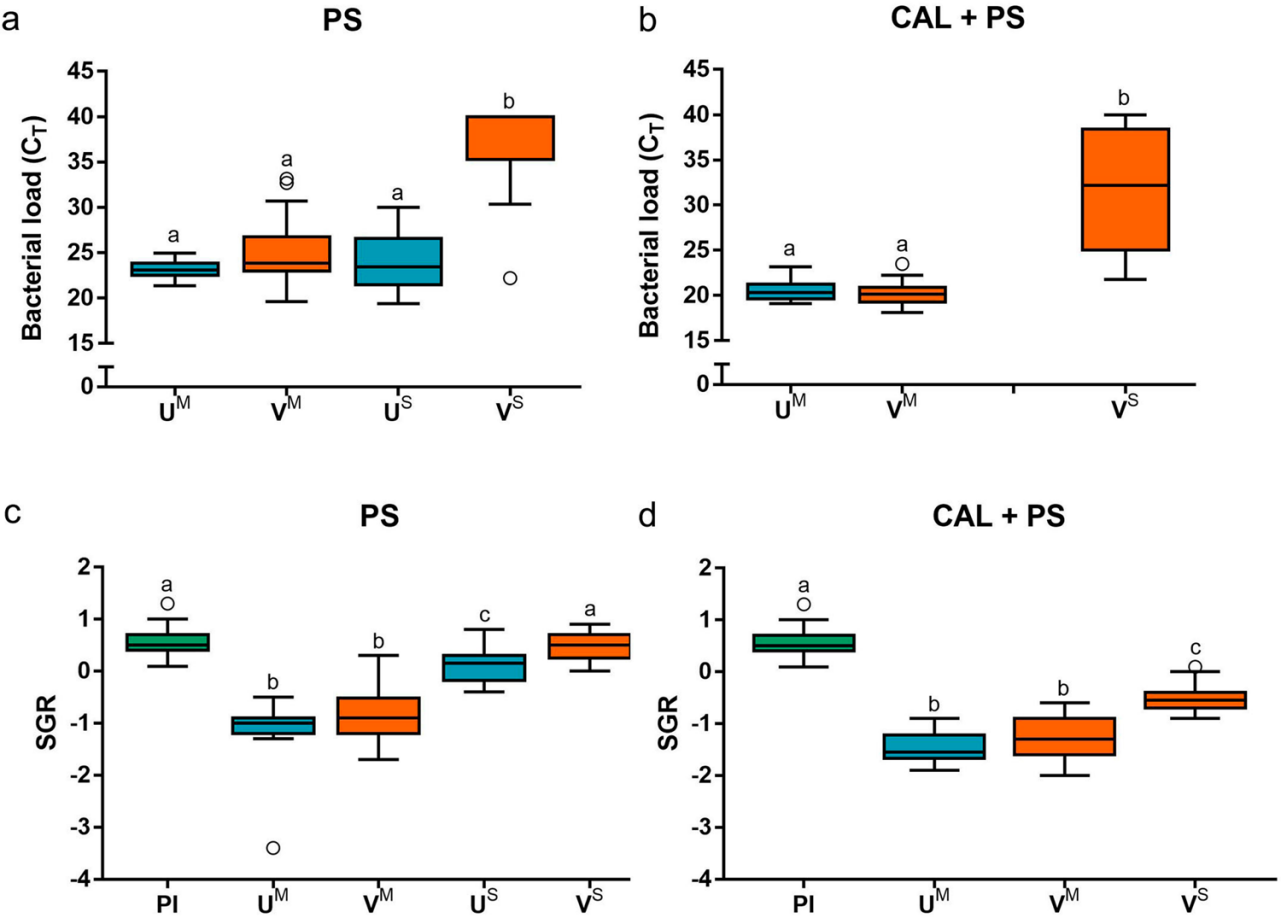
*Piscirickettsia salmonis* load and SGR in Atlantic salmon. Bacterial load (**a**,**b**) and SGR (**c**,**d**) in a single infection with *P. salmonis* and coinfection with *C. rogercresseyi* and *P. salmonis,* respectively, are shown. Due to the low number of surviving coinfected U^S^ fish, they were not included. Data represent mean ± SD. Statistical significance was obtained from the non-parametric Kruskal–Wallis test followed by a Dunn post-hoc test. *PS* single infected with *P. salmonis*; *CAL + PS* coinfected with *C. rogercresseyi* and *P. salmonis*; *PI* pre-infection; *U*^*M*^ unvaccinated moribund; *V*^*M*^ vaccinated moribund; *U*^*S*^ unvaccinated survivors; and *V*^*S*^ vaccinated survivors.

Bacterial load was compared between vaccinated and unvaccinated fish that survived the infection (V^S^ and U^S^, respectively) and those vaccinated and unvaccinated fish that were moribund (V^M^ and U^M^, respectively). The amount of *P. salmonis* was not significantly different between vaccinated and unvaccinated moribund fish, in neither single infection (PS) or coinfection (CAL + PS) treatments (Fig. 4a,b). On the other hand, vaccinated survivor fish (V^S^) showed the lowest bacterial load, both in a single (*p* < 0.01) and coinfection (*p* < 0.01). The effects of population and sex of fish were evaluated and did not significantly influence bacterial load (Fig. S4a,b online).

### Comparison of growth performance

Growth is a good physiological indicator of the health status of fish, so we compared the specific growth rate (SGR) between the four groups defined previously (V^S^, U^S^, V^M^, and U^M^). As expected, almost all infected fish showed a lower SGR than those observed before to infection (Fig. 4c,d). The only exception to this was the V^S^ fish group, exposed to a single infection, which showed no significant difference in comparison to the pre-infection group (PI, Fig. 4c), revealing a remarkable ability of V^S^ fish to prevent the harmful effects on growth of acute infection. In general, survivor fish (V^S^ and U^S^) showed a higher SGR than the moribund fish (V^M^ and U^M^) (Fig. 4c,d). The coinfection treatment was highly detrimental to the fish, with all the groups showing less growth than without coinfection (Fig. 4d). Similar to what was observed in the bacterial load study, we did not find effects of population or sex of fish on SGR (Fig. S4c,d online).

### Comparison of clinical signs

Our next objective was to compare clinical signs through a blind macroscopic examination in different tissues and organs in individual fish. Due to the high mortality of the unvaccinated groups, it was not possible to have sufficient fish to report clinical signs on the unvaccinated survivors group (n < 5), thus further comparison was carried out exclusively between three groups: V^S^, V^M^ and U^M^ fish (Tables 3 and 4). Differences between V^S^ and V^M^ exposed to a single infection of *P. salmonis* were found in 3 of 10 clinical signs: gill mucus, skin congestion, and gill paleness. V^S^ fish showed a higher incidence than V^M^ fish of gill mucus and skin congestion, but a lower incidence of gill paleness. In general, single infected moribund fish showed similar clinical signs. We only observed significant differences in skin congestion that was mostly present in V^M^ than in U^M^ and white hepatic nodules that were lower in V^M^ than U^M^ (Table 3). Differences between coinfected (CAL + PS) V^S^ and V^M^ fish were found in 2 of 10 clinical signs: gill mucus, and white hepatic nodules, with a high incidence in V^M^ than V^S^ (Table 4). Interestingly, in coinfection, both U^M^ and V^M^ fish did not show significant differences in any of the clinical signs analyzed (Table 4).

**Table 3:** Differences in clinical signs of vaccinated and unvaccinated moribund and vaccinated survivors Atlantic salmon single infected with *P. salmonis*.

Type of lesion or alteration	Presence of alterations	Number of fish			Proportion			X-squared U^M^ − V^M^	X-squared V^M^ − V^S^	df	*p*-value^a^ U^M^ − V^M^	*p*-value^a^ V^M^ − V^S^
		U^M^	V^M^	V^S^	U^M^	V^M^	V^S^					
Skin congestion	No	15	5	14	0.75	0.25	0.70	8.1	6.416	1	0.0044	0.01131
	Yes	5	15	6	0.25	0.75	0.30					
Desquamation	No	15	8	12	0.75	0.40	0.60	3.6829	0.9	1	0.0549	0.3428
	Yes	5	12	8	0.25	0.60	0.40					
Skin ecchymosis	No	20	20	18	1.00	1.00	0.90	0	0.52632	1	1	0.4682
	Yes	0	0	2	0.00	0.00	0.10					
Gill paleness	No	5	12	3	0.75	0.60	0.15	3.6829	5.1042	1	0.0549	0.02387
	Yes	15	8	17	0.25	0.40	0.85					
Gill mucus	No	2	2	15	0.10	0.10	0.75	0	14.731	1	1	0.000124
	Yes	18	18	5	0.90	0.90	0.25					
Gill melanomacrophagues	No	8	12	9	0.40	0.60	0.45	0.3428	0.401	1	0.3428	0.5266
	Yes	12	8	11	0.60	0.40	0.55					
White hepatic nodules	No	4	15	19	0.20	0.75	0.95	10.025	1.7647	1	0.0015	0.184
	Yes	16	5	1	0.80	0.25	0.05					
Hepatomegaly	No	20	20	19	1.00	1.00	0.95	0	0	1	1	1
	Yes	0	0	1	0.00	0.00	0.05					
Spleen paleness	No	0	0	0	0.00	0.00	0.00	0	0	1	1	1
	Yes	20	20	20	1.00	1.00	1.00					
Splenomegaly	No	0	0	1	0.00	0.00	0.05	0	0	1	1	1
	Yes	20	20	19	1.00	1.00	0.95					

U: unvaccinated; V: vaccinated; M: moribund; and S: survivors.

^a^*p*-values were obtained from non-parametric chi-square test to compare proportions.

**Table 4:** Differences in clinical signs of vaccinated and unvaccinated moribund and vaccinated survivors Atlantic salmon coinfected with *C. rogercresseyi* and *P. salmonis*.

Type of lesion or alteration	Presence of alterations	Number of fish			Proportion			X-squared U^M^ − V^M^	X-squared V^M^ − V^S^	df	*p*-value^a^ U^M^—V^M^	*p*-value^a^ V^M^—V^S^
		U^M^	V^M^	V^S^	U^M^	V^M^	V^S^					
Skin congestion	No	4	5	1	0.20	0.25	0.05	0	1.7647	1	1	0.184
	Yes	16	15	19	0.80	0.75	0.95					
Desquamation	No	4	4	5	0.20	0.20	0.25	0	0	1	1	1
	Yes	16	16	15	0.80	0.80	0.75					
Skin ecchymosis	No	11	9	8	0.55	0.45	0.40	0.1	0	1	0.7518	1
	Yes	9	11	12	0.45	0.55	0.60					
Gill paleness	No	0	0	1	0.00	0.00	0.05	0	0	1	1	1
	Yes	20	20	19	1.00	1.00	0.95					
Gill mucus	No	0	0	11	0.00	0.00	0.55	0	12.539	1	1	0.0003985
	Yes	20	20	9	1.00	1.00	0.45					
Gill melanomacrophagues	No	5	4	10	0.25	0.20	0.50	0	2.7473	1	1	0.09742
	Yes	15	16	10	0.75	0.80	0.50					
White hepatic nodules	No	0	1	12	0.00	0.05	0.60	0	11.396	1	1	0.000736
	Yes	20	19	8	1.00	0.95	0.40					
Hepatomegaly	No	17	18	19	0.85	0.90	0.95	0	0	1	1	1
	Yes	3	2	1	0.15	0.10	0.05					
Spleen paleness	No	0	0	0	0.00	0.00	0.00	0	0	1	1	1
	Yes	20	20	20	1.00	1.00	1.00					
Splenomegaly	No	1	0	0	0.05	0.00	0.00	0	0	1	1	1
	Yes	19	20	20	0.95	1.00	1.00					

*U* unvaccinated; *V* vaccinated; *M* moribund; and *S* survivors.

^a^*p*-values were obtained from non-parametric chi-square test to compare proportions.

## Discussion

Vaccination has been a successful strategy to prevent or reduce the severity of some infectious diseases in farmed fish^10,20^. In salmon aquaculture, vaccination entails a significant part of the health production cost and has certainly contributed to the control of different bacterial and viral diseases around the world, including furunculosis, infectious hematopoietic necrosis (IHN), and edwardsiellosis among others^21–26^. However, the reasons why vaccines fail to control other diseases like piscirickettsiosis remains unknown^9,12,27,28^. In this study we report, for two populations of Atlantic salmon, that between-familiar variation is a strong intrinsic factor that controls the success of vaccination in protecting against *P. salmonis*. Vaccines against *P. salmonis* have been available since 1999^29^, and the reported efficacy from laboratory and field experiments has varied from 95 to 49%^30–33^. Failure to protect a large amount of fish in production conditions due to between-familiar variation may break the herd immunity benefit^34^ and reduce the efficacy of *P. salmonis* vaccines. More studies are necessary to evaluate if variation in the host immune response to vaccination could explain between-familiar differences in resistance observed in vaccinated fish.

Recently, the selection of resistant fish in a non-vaccinated environment has been proposed and implemented as a complementary strategy for vaccination to prevent piscirickettsiosis^35^. Natural resistance against *P. salmonis* in Atlantic salmon seems to be polygenic^36^ and partially related to an iron deprivation mechanism that robs bacteria of nutrients^37^. However, our results show that natural resistance and vaccine-mediated resistance do not seem to act synergistically, as was previously assumed. A similar phenomenon was observed by Drangsholt et al. (2011) for Furunculosis, another important disease in Atlantic salmon^38^. This disease is caused by *Aeromonas salmonicida*, a facultative intracellular bacteria that, similar to *P. salmonis*, has the ability to infect and replicate in host cells in order to bypass the host defense mechanisms^39,40^. Thus, similar to Drangsholt et al., our results indicate that not all resistant fish selected in an environment without vaccination will be resistant following vaccination^38^. This could negatively impact current vaccination success, since fish farmers usually use a few “elite” males to fertilize millions of eggs in production conditions, therefore, this needs to be studied in more depth. Alternatively, the host genetic variation could be used in selective breeding to increase the efficacy of vaccines against *P. salmonis*, as has been proposed for terrestrial animals^41,42^ and successfully applied in livestock^43,44^.

Age is another intrinsic factor that has been suggested as a determinant of low efficacy of vaccines, which, since fish are ectotherms, is usually measured as degree-days. In Atlantic salmon, it has been reported that the maximum concentration of IgM antibodies against *P. salmonis* occurs at 600–800 degree-days after vaccination (60–80 days to 10 °C), rapidly decreasing after 1,300 degree-days^33^. Atlantic salmon reach a commercial size between 3,000 to 6,000 degree-days after vaccination, depending on the strain and other factors. Currently, we do not know how long vaccine protection lasts in resistant fish, or if resistance is correlated with time of protection due to antibodies induced by vaccination.

We have previously shown that coinfection of sea lice is a strong extrinsic factor that explains lower disease resistance in Atlantic salmon^45^ and reduced efficacy of vaccines against *P. salmonis*^18^. Sea lice have the most significant economic impact of any parasite on the global salmon farming^46^, due to detrimental effects on survival, growth and flesh quality, in addition to increased susceptibility to secondary infections due to epithelial damage that also induces high levels of stress^46–48^. Infection with *C. rogercresseyi* alone has been reported to decrease Th1 responses, macrophage activation, TLR-mediated responses, and iron regulation^49–51^. In contrast, *P. salmonis* has been shown to both imbalances the host innate immune response^28^ and downregulated genes involved in the adaptive immune response in infected fish as a means to survive, replicate, and escape host defense^11^. However, despite this complex scenario, we find that there is a substantial phenotypic and genetic variation in the resistance of coinfected and vaccinated fish. For this reason, we conclude that resistance in a vaccinated and coinfected environment is also a heritable trait and therefore is suitable for genetic improvement.

Correlation of resistance to single infection with *P. salmonis* and coinfection with *Caligus* was previously reported as zero in a study with unvaccinated fish^45^; however, in this study, our correlation estimates were very high and positive in the unvaccinated environment. We propose that this difference is due to a combination of up to three factors: First, the low number of families evaluated in the previous work (19 full-sib families from one population) in comparison with this study (200 full-sib families from two populations); Second, in this study infection was by intraperitoneal injection instead of cohabitation, which may change the immune response to infection with *P. salmonis*^52^; Third, in this study, we used sea lice as the primary pathogen. Since the host immune response has only been analyzed against *P. salmonis* or *C. rogercresseyi* separately^51,53^, we do not know if coinfection in this order may have affected the results. Thus, we conclude that resistance against *P. almonis* and resistance to coinfection when the route of infection was intraperitoneal and *Caligus* was the primary pathogen, are controlled by similar loci.

In this study, resistance and susceptibility were evaluated by bacterial load, growth, and clinical signs associated with this disease. Our results showed that vaccinated survivors had a lower bacterial load than vaccinated moribund fish and unvaccinated fish (survivors and moribund). Thus, vaccinated survivors but not vaccinated moribund fish were able to clear bacteria and thus decrease bacterial load after *P. salmonis* challenge^15^. Furthermore, regardless of whether the fish were vaccinated or not, all moribund fish demonstrated decreased growth rates. Concerning clinical signs, skin congestion and peeling were mostly present in vaccinated fish infected by *P. salmonis*, so we can infer that these are side effects of the vaccine following infection. The most characteristic macroscopic lesion of *P. salmonis* infection is the presence of multifocal subcapsular hepatic nodules^54^, which we found more often in unvaccinated than in vaccinated fish in this study. Thus, we conclude that vaccination reduces some clinical signs of piscirickettsiosis in the liver, but it may not reduce the spread of infection or mortality, which can lead to an incorrect anatomopathological diagnosis in vaccinated fish.

In conclusion, the current study demonstrates that host genetic variation is a strong intrinsic factor that determines the efficacy of the vaccines against the bacterial pathogen *P. salmonis* in Atlantic salmon. This may explain why vaccines currently provide variable protection against piscirickettsiosis, partially protecting some hosts while leaving others unprotected. In addition to the overriding effect of sea lice infection on vaccination^18^, between-family variation may further decrease the efficacy of *P. salmonis* vaccines in the field, which has caused the high use of antibiotics in this industry. Considering these factors in the development of new vaccines and the selective breeding of fish may help mitigate the economic and environmental impact of this disease.

## Materials and methods

### Fish and vaccines

Two pedigree populations of Atlantic salmon (*Salmo salar*) called Fanad and Lochy were used in this study^18^ (Table 1). The two populations were managed separately and had different origins. Fish were provided in 2016 by the salmon fish farming company Salmones Camanchaca and pit tagged in April 2016 at an average weight of 26.4 ± 3.9 g and 30.2 ± 4.2 g, for populations Fanad and Lochy, respectively. During the freshwater growth period, salmon were immunized twice using commercial vaccines, following the strict Salmones Camanchaca protocols. First, fish were vaccinated by intraperitoneal (IP) injection with a pentavalent vaccine against *P. salmonis, Vibrio ordalii, A. salmonicida*, IPNV (infectious pancreatic necrosis virus) and ISAV (infectious salmon anemia virus). Second, fish were immunized by IP injection against *P. salmonis* using a monovalent live attenuated vaccine at the same time as the first vaccination. Since 2016, this double vaccination strategy has been a common practice in the Chilean salmon industry^17^. Fish were transferred as smolts to the Aquadvice experimental station in Puerto Montt, Chile. Unvaccinated fish were injected with PBS (phosphate-buffered saline) and used as control (Table 1). Prior to transferring the fish, a health check by RT-PCR was performed to verify that the fish were free of viral (ISAV and IPNV) and bacterial pathogens (*Vibrio* sp., *Flavobacterium* sp., *P. salmonis,* and *Renibacterium salmoninarum*). At the experimental station, all fish underwent a 15 days acclimatization period in seawater (salinity of 32% and a temperature of 15 ± 1 °C). Fish were fed daily ad libitum with a commercial diet.

### Calculation of *Piscirickettsia salmonis* LD_50_

The median lethal dose (LD_50_) of *P. salmonis* (EM-90 type) was determined as previously described^18^. Briefly, animals from both populations were distributed in eight tanks of 350 L (n = 60 fish per tank). The LD_50_ was calculated in fish infected by IP injection with 200 μL of a *P. salmonis* suspension. Three dilutions were assessed from stock with concentrations of 1 × 10^6.63^ TCID/mL (TCID = median tissue culture infective dose): 1 × 10^–3^ TCID/mL, 1 × 10^–4^ TCID/mL, and 1 × 10^–5^ TCID/mL. Controls were injected with 200 μL of PBS. Fish were monitored daily for 30 days, and mortalities were recorded. The presence of bacteria was assessed by qRT-PCR. In both infection scenarios, a single infection and coinfection, the highest dose of *P. salmonis* was used (1 × 10^–3^ TCID/mL) as a conservative measure because the fish grow about 100 g between LD_50_ and the main challenge (50 days).

### Infection design, trait of resistance and protection added by vaccine

Fish were treated with two different types of infection, a single infection with *P. salmonis* (PS) or coinfection with both *C. rogercresseyi* and *P. salmonis* (CAL + PS) as previously described^18^. In short, infections against *P. salmonis* occurred at 822 ATU (accumulated thermal units) within the immunization period described by the vaccine manufacturer. Vaccinated and unvaccinated fish from populations Fanad and Lochy were equally distributed in four tanks of 6 m^3^, with two replicates per type of infection. For the single infection with *P. salmonis*, fish were IP injected. For the coinfection, fish were exposed first to sea lice and then to *P. salmonis*. A coinfection procedure was established based on our previous experience with this study model^45,55^. Infections with sea lice were performed by adding 60 copepodites per fish to each tank of coinfection. Copepodites were collected from egg-bearing females reared in the laboratory and confirmed as “pathogen-free” (*P. salmonis*, *R. salmoninarum,* IPNV, and ISAV) by RT-PCR diagnostic. After the addition of parasites, water flow was stopped for a period of 8 h, and tanks were covered to decrease light intensity, which favors a successful settlement of sea lice on fish^55^. A placebo procedure was applied to single infection tanks, keeping them in darkness and controlling the volume of water, temperature, oxygen levels, and fish density equivalent to those that were measured in coinfected tanks^18^. The secondary infection was performed with *P. salmonis* after seven days of sea lice infestation, and the establishment of the parasites was confirmed and quantified on all fish. Therefore, our experimental design had two types of treatments: (1) single infection (PS) or coinfection (CAL + PS); and (2) vaccinated or unvaccinated fish. Vaccinated and unvaccinated fish with a single infection were distributed in tanks 1 and 2, and vaccinated and unvaccinated fish with a coinfection were distributed in tanks 3 and 4. Further, fish were fasted for one day prior to each procedure to minimize the detrimental effects of stress on water quality parameters. Finally, fish were sedated with AQUI-S (50% Isoeugenol, 17 mL/100 L water) to reduce stress during handling. Fish were monitored daily for 30 days, and resistance to *P. salmonis* was measured individually as days to death. Protection added by vaccination was calculated as the difference of resistance between vaccinated fish and their unvaccinated full-sibs and represented under a single Genetic and Environment model (GxE, G = full-sib family; E = Vaccination treatment).

### Comparison of moribund and survivor fish

Bacterial load, growth, and macroscopic lesions were evaluated in survivors and moribund fish. Moribund fish were obtained as dying fish when 50% of mortality was reached in both a single infection and coinfection treatments. Moribund fish were recognized and collected by three behavioral traits: lethargy, no response to stimuli, and slow swimming close to the tank wall. Resistance to *P. salmonis* was measured by days to death and mortality (alive versus dead) and monitored for 30 days^15,45^, survivors fish comprised those that lived at the end of experiment^15^. Forty fish were collected from each group of moribund and survivors, and from each treatment (PS and CAL + PS) and comparisons were performed between unvaccinated and vaccinated fish, twenty fish each group. However, due to the low number of unvaccinated survivors fish coinfected with *P. salmonis* and sea lice, it was not possible to compare with the vaccinated survivors fish.

### Specific growth rate (SGR)

SGR was evaluated for moribund and survivors fish. The specific growth rate was calculated previous to infection, and post-infection as SGR = ((*ln*w2 − *ln*w1)*t^−1^)*100, where w2 corresponds to final weight, w1 to the initial weight, and t corresponds to the number of days between infection and death of the fish or the end of the trial if they survived^56^.

### *Piscirickettsia salmonis* load

*Piscirickettsia salmonis* load was evaluated for moribund and survivors fish. *P. salmonis* load was estimated based on the amount of specific ribosomal RNA from the bacteria in the head kidneys of the infected fish, as measured by qRT-PCR. Dead fish were not used to evaluate bacterial load. Threshold cycle (C_T_) values from bacterial RNA was used as an indication of the bacterial load as previously described^18^. Head kidney samples were extracted from 20 moribund and survivors fish per group and preserved in RNAlater at − 80 °C until RNA extraction. RNA was extracted from tissue samples with the TRIzol reagent (Thermo Fisher Scientific, MA, USA) following the instructions provided by the manufacturer. DNA was removed through an additional step using a DNase incubation for 60 min at 37 °C. The quality of the RNA extraction was checked by visualizing the 28S and 18S rRNA bands resolved in 1% of agarose gels stained with SYBR Safe DNA gel stain (Invitrogen, CA, USA), and the total concentration of the RNA was measured spectrophotometrically in a MaestroNano device (MAESTROGEN, Hsinchu, Taiwan). One hundred nanograms of purified total RNA was used for the qRT-PCR reactions. The qRT-PCR reaction was prepared using the Brilliant III SYBR master mix (Agilent Technologies, CA, USA) by adding the template RNA, probes, and primers as described previously^57^. qRT-PCR was performed in the Eco Real-Time PCR system (Illumina, CA, USA), whose results were expressed in terms of C_T_. All samples were tested in triplicates and were calibrated to a plate standard that contained a combination of samples from all groups tested. Primers used for 23S gene of *S. salar* were forward primer TCTGGGAAGTGTGGCGATAGA and reverse primer TCCCGACCTACTCTTGTTTCATC.

### Necropsy analysis

Macroscopic lesions from 20 fish per treatment were analyzed on moribund and survivors fish^13^; almost all survivors sampled fish were vaccinated, except one unvaccinated fish that survived to *P. salmonis* infection (data not shown). Fresh samples were analyzed by two veterinarians who were blinded to the treatments. Macroscopic lesions evaluated in the tissues were peeling or undergoing desquamation, congestion, and ecchymosis in the skin, paleness, and melanomacrophages in the gills, white hepatic nodules, hepatomegaly, spleen paleness, and splenomegaly. Macroscopic lesions were indicated as present or absent.

### Statistical analysis

Significance levels of resistant to *P. salmonis* were obtained using a two-way ANOVA followed by a Tukey post-hoc test and unpaired t-test. The effects of populations and sex of fish on SGR and *P. salmonis* load were analyzed using a non-parametric Kruskal–Wallis test followed by a Dunn post-hoc test. Additionally, differences in the clinical signs of the *P. salmonis* infection between different treatments were analyzed using a non-parametric Chi-square proportion. All statistical analyses were performed using R Core Team (RStudio, Vienna, Austria). Graphs were designed with GraphPad Prism 8.0 software (GraphPad Software, CA, USA).

### Quantitative genetic analysis

Each population in this study has a different genetic origin and has been managed as closed populations during the domestication process. Thus, (co) variance components of days to death were estimated independently for each population from the data of its genealogy (Table 1) using VCE 6.0 software by Groeneveld et al.^58^.

Heritability of days to death was estimated using the following univariate animal model:

$$y = \upsilon 1 \, + X_{1} t + X_{2} i + X_{3} v + Za + e,\,\,\,{\text{Model}}\,1$$

where *y* is the vector of the trait days to death, *μ* is the overall mean effect*, ****t*** is the fixed effect of tank; ***i*** is the fixed effect of type of infection; ***v*** is the fixed effect of group of vaccination; ***a*** is the random effects vector of animal effects, with ***a*** ~ N(**0**, *σ*_*a*_^2^***A***); and ***e*** is the random vector of errors, with ***e*** ~ N(**0**, *σ*_*e*_^*2*^***I***_***e***_). ***X***_***1***_, ***X***_***2***_, ***X***_***3,***_ and ***Z*** are incidence matrices, and ***A*** is the numerator relationship matrix obtained from pedigree information. The magnitude of estimated heritability was established following the classification of Cardellino and Rovira^59^: low (0.05–0.15), medium (0.20–0.40), and high (0.45–0.60) and very high (> 0.65).

Genotype–environment interactions (GxE) were estimated by means of genetic correlations between the trait days to death measured in one environment (i.e., unvaccinated and single infection with *P. salmonis*) and the same trait measured in the other environment (i.e., vaccinated and coinfection).

Genetic correlations were estimated using the following bivariate animal model:

$$y_{1} ,\;y_{2} = X_{1} d + X_{2} t(d) + X_{3} i(d) + X_{4} v(d) + Za(d) + e,\,\,\,{\text{Model}}\,1$$

where, ***y***_***1***_ and ***y***_***2***_ are the data vectors for the traits of interest (days to death in vaccinated and unvaccinated fish); ***d*** is the fixed vector of trait effects; ***t(d)***, ***i(d)***, ***v(d),*** are the fixed effects of tank, type of infection and group of vaccination effects within trait, respectively; ***a(d)*** is the random vector of animal effects within trait, with ***a(d)*** ~ N(***0***, ***A*** ⊗ ***G***); and ***e*** is the random vector of errors, with ***e*** ~ N(***0***, ***I*** ⊗ ***R***). The matrix ***G*** is a 2 × 2 variance–covariance matrix between traits defined by a genetic additive correlation term, *r*_*g*_, and a genetic variance (*σ*_*gj*_^2^) for each trait. The matrix ***R*** is an unstructured 2 × 2 residual variance–covariance matrix with a different variance for each trait (*σ*_*ej*_^2^), and a covariance between traits (*σ*_*eij*_). All other terms were previously defined. Correlations were classified as low (0–0.39), medium (0.40–0.59), high (0.60–0.79), and very high (0.80–1), regardless whether it was positive or negative. Significance testing of the estimates of heritability and genetic correlation were approximate as suggest by Åkesson et al.^60^. Thus, any genetic parameter value was considered significantly different from zero with P < 0.05 or P < 0.01 when the absolute value of the estimate was more than twice or three times the standard error, respectively.

### Ethics statement

This study was carried out in accordance with the guide for the care and use of experimental animals of the Canadian Council on Animal Care. The protocol was approved by the Bioethics Committee of the Pontificia Universidad Católica de Valparaíso and the Comisión Nacional de Investigación Científica y Tecnológica de Chile (FONDECYT N° 1140772). The animals were anesthetized with benzocaine prior to each handling process. Euthanasia was performed using an overdose of anesthesia. All efforts were made to minimize animal stress and to ensure that termination procedures were efficiently performed.

## Supplementary information

Supplementary file1
