# Supplementary material for: Host genetic variation explains reduced protection of commercial vaccines against Piscirickettsia salmonis in Atlantic salmon
Source: Sci Rep. 2020 Oct 26;10:18252. doi: 10.1038/s41598-020-70847-9 (PMC7588420; doi:10.1038/s41598-020-70847-9)
Supplement: Supplementary file 1 — Supplementary file1 [file 41598_2020_70847_MOESM1_ESM.docx]

**Supplementary Information**

**Host genetic variation explains reduced protection of commercial vaccines against *Piscirickettsia salmonis* in Atlantic salmon**

Carolina Figueroa^1^, Pamela Veloso^1,2^, Lenin Espin^3^, Brian Dixon^4^, Débora Torrealba^5^, Juan Manuel Afonso^3^, Carlos Soto^6^, Pablo Conejeros7, José A. Gallardo^1^.

^1^Escuela de Ciencias del Mar, Pontificia Universidad Católica de Valparaíso, Altamirano 1424, Valparaíso, Región de Valparaíso, postal code: 2360007, Chile.

^2^ Programa de Doctorado en Ciencias, mención Recursos Naturales Acuáticos,

Universidad de Valparaíso, Blanco 951, Valparaíso, Región de Valparaíso, postal code: 2391415, Chile.

^3^Facultad de Ciencias del Mar, Universidad de Las Palmas de Gran Canaria, 35017 Tafira Baja, Las Palmas, postal Code: 35017, Spain.

^4^Department of Biology, Faculty of Science, University of Waterloo, 200 University Ave W, Waterloo, ON N2L 3G1, Canada.

^5^Department of Agricultural, Food & Nutritional Science, University of Alberta, 116 St & 85 Ave, Edmonton, AB T6G 2R3, Canada.

^6^Salmones Camanchaca Diego Portales 2000, Puerto Montt, postal code: 5503642, Chile.

^7^Centro de Investigación y Gestión de Recursos Naturales (CIGREN), Facultad de Ciencias, Universidad de Valparaíso, Blanco 951, Valparaíso, Región de Valparaíso, postal code: 2391415, Chile.


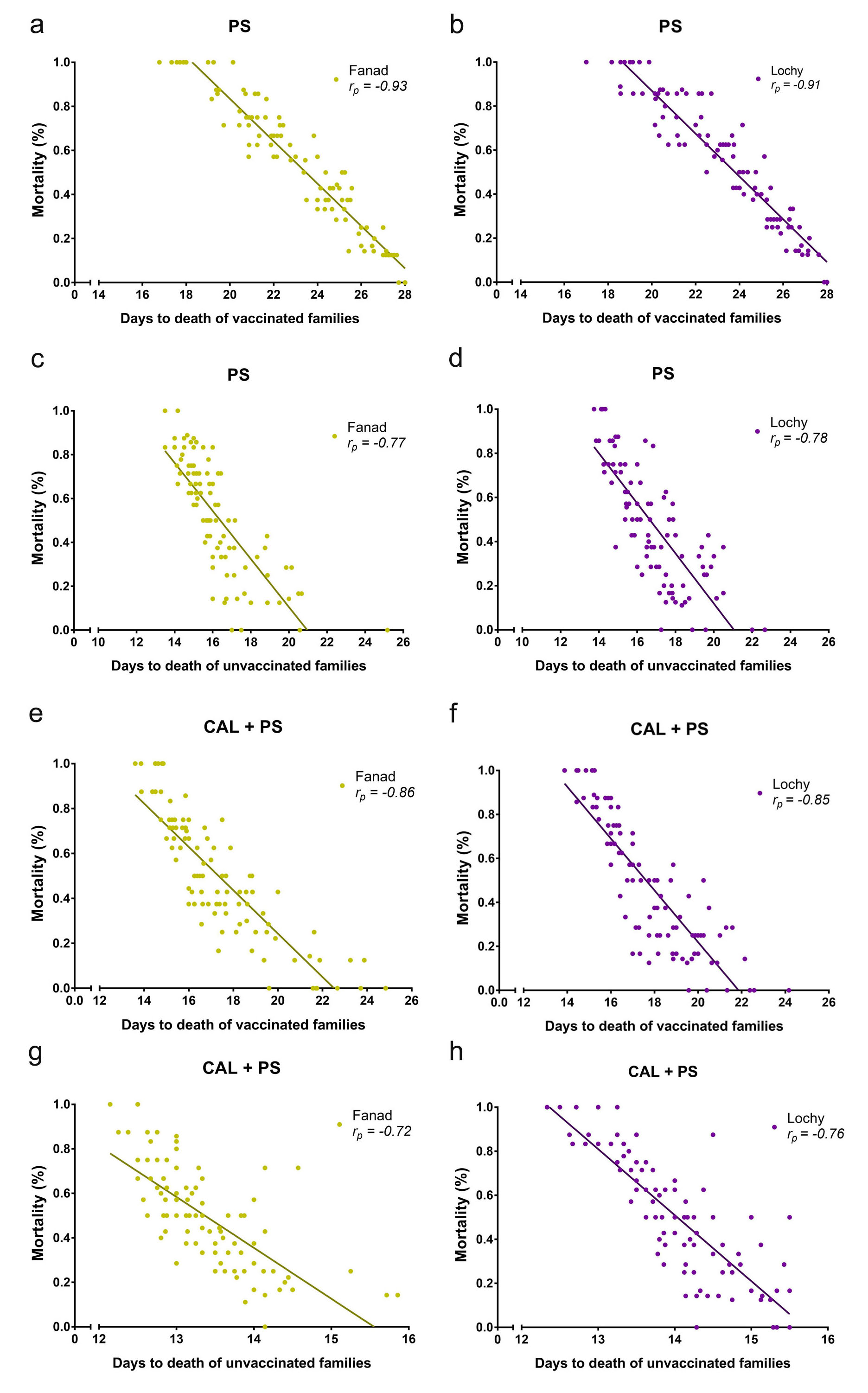


**Figure S1.** Phenotypic correlation between days to death and mortality. Correlation for vaccinated fish families (a-b-e-f) and unvaccinated fish families (c-d-g-h) of the populations Fanad (yellow) and Lochy (purple) that were exposed to single infection with *P. salmonis* (PS) or coinfection with *C. rogercresseyi* and *P. salmonis* (CAL + PS).

**
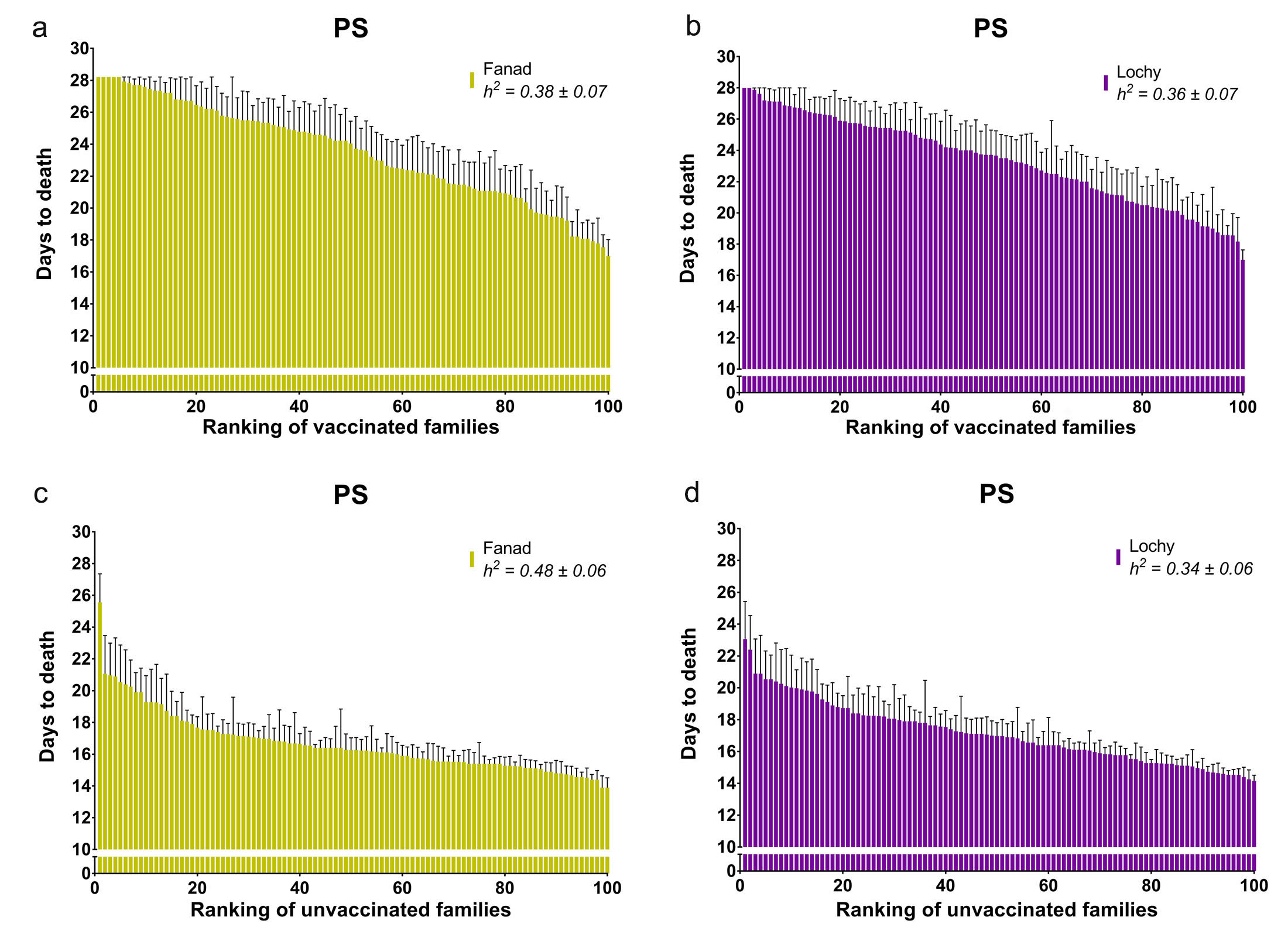
**

**Figure S2.** Variation of family resistance to *P. salmonis* in a single infection. Genetic resistance against *P. salmonis* as days to death in vaccinated (a-b) and unvaccinated (c-d) fish of the populations Fanad (yellow) and Lochy (purple) that were exposed to single infection with *P. salmonis* (PS).

**
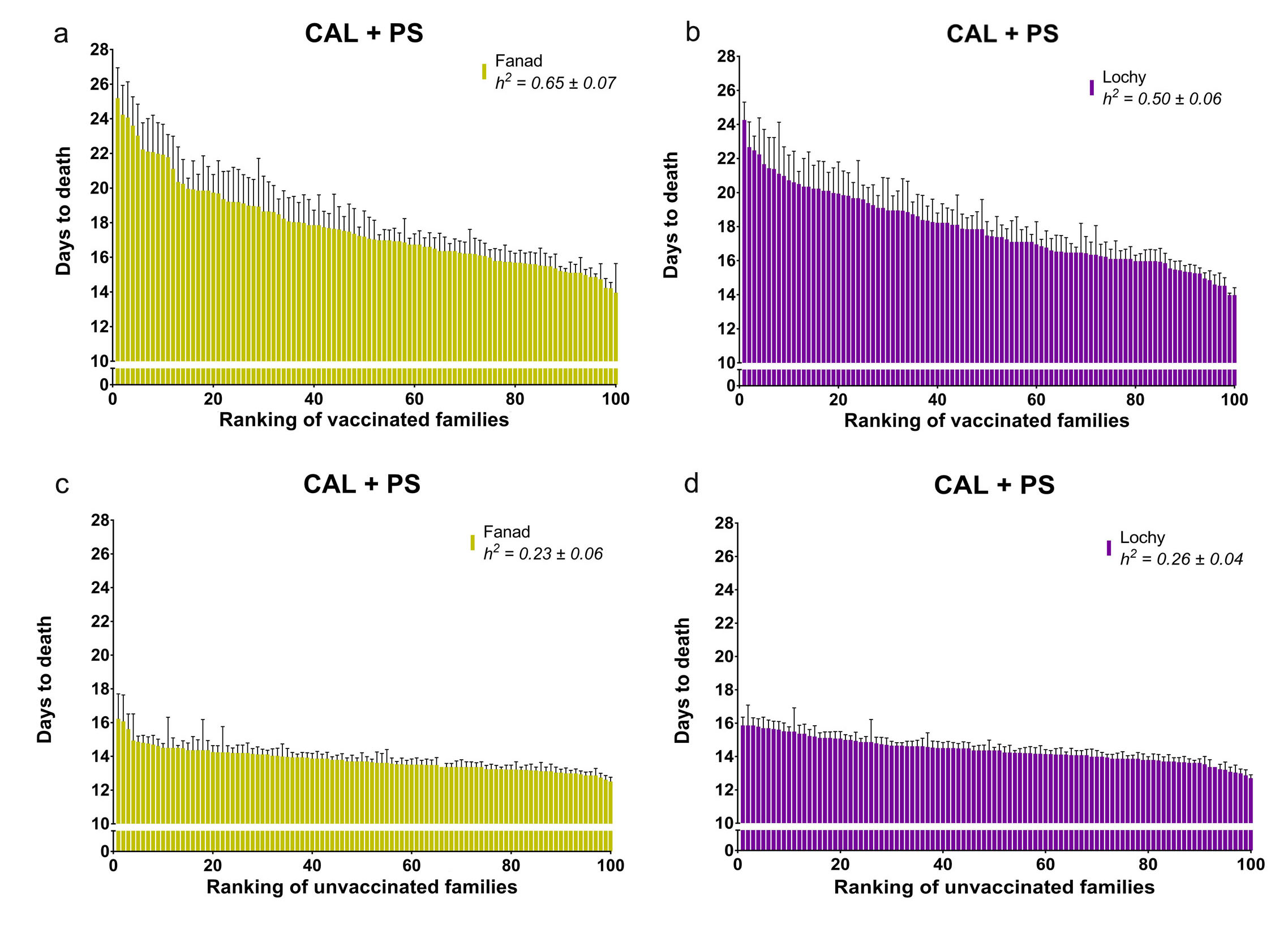
**

**Figure S3.** Variation of family resistance to *P. salmonis* in coinfection. Genetic resistance against *P. salmonis* as days to death in vaccinated (a-b) and unvaccinated (c-d) fish of the populations Fanad (yellow) and Lochy (purple) that were exposed to coinfection with *C. rogercresseyi* and *P. salmonis* (CAL + PS).

**
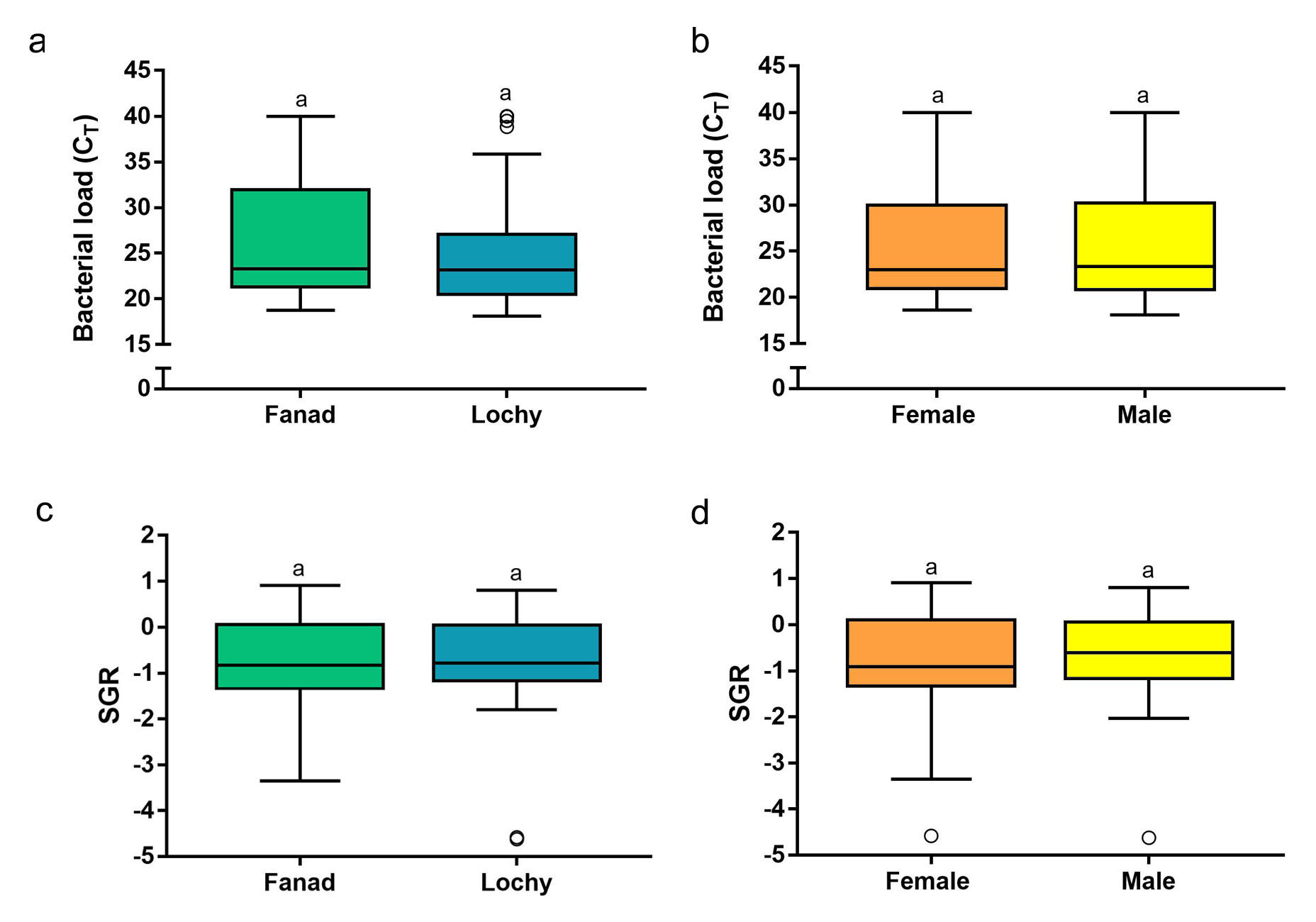
**

**Figure S4.** Comparison of *P. salmonis* bacterial load (C_T_ value) between populations (a) and sex of fish (b), and comparison of SGR between populations (c) and sex of fish (d). Data represent mean ± SD. Statistical significance was obtained from the non-parametric Kruskal-Wallis test followed by a Dunn post-hoc test.
